# The Opportunities and Challenges of Integrating Population Histories Into Genetic Studies for Diverse Populations: A Motivating Example From Native Hawaiians

**DOI:** 10.3389/fgene.2021.643883

**Published:** 2021-09-27

**Authors:** Charleston W. K. Chiang

**Affiliations:** ^1^Department of Population and Public Health Sciences, Center for Genetic Epidemiology, Keck School of Medicine, University of Southern California, Los Angeles, CA, United States; ^2^Department of Quantitative and Computational Biology, University of Southern California, Los Angeles, CA, United States

**Keywords:** population genetics, human genetics, genome-wide association studies, natural selection, Native Hawaiians, demographic history

## Abstract

There is a well-recognized need to include diverse populations in genetic studies, but several obstacles continue to be prohibitive, including (but are not limited to) the difficulty of recruiting individuals from diverse populations in large numbers and the lack of representation in available genomic references. These obstacles notwithstanding, studying multiple diverse populations would provide informative, population-specific insights. Using Native Hawaiians as an example of an understudied population with a unique evolutionary history, I will argue that by developing key genomic resources and integrating evolutionary thinking into genetic epidemiology, we will have the opportunity to efficiently advance our knowledge of the genetic risk factors, ameliorate health disparity, and improve healthcare in this underserved population.

## Introduction

Genome-wide association studies (GWASs) have revealed the polygenic nature of human complex traits and diseases (Hirschhorn and Daly, 2005; McCarthy et al., 2008; Visscher et al., 2017), but these successes are heavily biased toward European-ancestry populations (Need and Goldstein, 2009; Popejoy and Fullerton, 2016; Spratt et al., 2016). To truly personalize medicine for everyone, we need to better understand both environmental/lifestyle risk factors and the genetic etiology of complex diseases, particularly in geographically diverse, often underserved, populations. It remains a challenge to attain sample sizes from diverse populations comparable to existing European-ancestry cohorts (>1 million individuals). Even when genetic data from understudied populations are included, they often comprise a small contributing part of a larger consortium, thereby masking any population-specific effects. There is thus a need to broadly include diverse populations in genomic studies through focused efforts. Whereas consortium-scale sample sizes are required to detect individual variants with ever-decreasing effect sizes associated with a complex trait, the genetic contributions to phenotypic differences among populations result from the distinct population history and unique interactions with the environment of the past or the present, which can be learned from moderately sized studies. For understudied populations, the focus is therefore both to transfer knowledge gained from large-scale Euro-centric studies and to supplement our understanding with insights specific to the population at hand.

Genetic and phenotypic differences between populations can arise through two broad categories of evolutionary mechanisms: demographic events and natural selection. An example of demographic events is a population bottleneck. In a bottlenecked population, alleles with functional, deleterious, consequences can, by chance, overcome the impact of negative selection (Ohta, 1973) to reach higher frequencies and, in turn, explain a greater proportion of the heritability of a complex trait compared to alleles in a non-bottlenecked population (Lim et al., 2014; Lohmueller, 2014; Locke et al., 2019). An example of natural selection is local adaptation to selective pressures such as climate, diet, UV exposures, or pathogens (Fan et al., 2016; Mathieson, 2020; Rees et al., 2020). Alleles underlying adaptive traits will increase in frequency in the local population. But as the environment changed in modern societies, these adaptations could manifest as diseases and contribute to differences in genetic risk between populations (Greaves, 2007; Stearns et al., 2010; Fay, 2013). Leveraging these evolutionary events in practice has already identified population-enriched alleles disproportionately contributing to human complex traits in multiple populations around the globe (Zhernakova et al., 2010; Moltke et al., 2014; Sidore et al., 2015; Zoledziewska et al., 2015; Minster et al., 2016; Steri et al., 2017; Grarup et al., 2018a, b; Locke et al., 2019; Asgari et al., 2020; Lin et al., 2020). These discovered alleles are oftentimes rare and difficult to map in large continental populations, but were found using only a moderately sized (by GWAS standards) cohort. Therefore, a better understanding of our evolutionary past will enable better designs and interpretations of genetic epidemiology studies, provide an opportunity to better understand the biology of human traits and diseases, help explain the disparity in risks among populations today, and allow the incorporation of evolutionary insights into our clinical practice (Stearns et al., 2010). However, these questions have not been systematically investigated in geographically diverse populations around the globe.

As an illustrative and motivating example, I will describe the challenges and benefits to combine evolutionary insights and genetic studies with the Native Hawaiian population. Though they are one of the smallest ethnic minorities in the United States, consisting of 1.2 million individuals and 0.4% of the United States census in 2010, Native Hawaiians and other Pacific Islanders (alone or in combination with other races) showed the second fastest rate of growth at 40% between 2000 and 2010. Compared to European- or Asian-Americans, Native Hawaiians display alarming rates of obesity, diabetes, cardiovascular diseases, cancers, and other related chronic health conditions (Grandinetti et al., 2002; Pike et al., 2002; Maskarinec et al., 2009; Mau et al., 2009; Madan et al., 2012; Singh and Lin, 2013; Tung and Barnes, 2014; Braden and Nigg, 2016). Environmental and/or social factors undoubtedly play an important role for these disparity, but in some cases, the risks for diseases are elevated even after adjusting for BMI and other socioeconomic and lifestyle factors (Pike et al., 2002; Maskarinec et al., 2009; Madan et al., 2012; Singh and Lin, 2013). This suggests that systematic differences in the number, frequencies, or effects of genetic risk alleles could partly explain the differences in risk among populations. The history of Native Hawaiians exemplifies all major evolutionary mechanisms influencing the pattern of variations in humans – population size changes, adaptation, and recent admixture. I will describe the opportunities to leverage extensively characterized genetic history for understanding the Hawaiian-specific disease architecture, current challenges that inhibit large-scale and systematic genetic studies, and important considerations of partnering with Native Hawaiians to perform genetic research. While I focus on leveraging evolutionary insight to improve the design and interpretation of genomic studies in understudied populations, there are important ethical considerations of studies with indigenous communities. I describe briefly my own experience and approach, and note that a large body of literature exists (e.g., Claw et al., 2018; Merriman and Wilcox, 2018; Garrison et al., 2019; Fox, 2020; Hudson et al., 2020, among others) that could not be covered in detail here. Finally, the opportunities and challenges described here are not limited to Native Hawaiians and are generally applicable to other understudied populations around the globe.

## Demographic and Admixture History of Native Hawaiians

There is no detailed characterization of the demographic history of Native Hawaiians using genetic data, though there are suggested models for Eastern Polynesians based on archeological findings, ancient and modern DNA studies, and oral history. Because of the shared genetic ancestry with aboriginal people in Island Southeast Asia, it has been hypothesized that Austronesian-speaking people from locations such as Taiwan or the Philippines migrated to the remote reaches of Oceania and Western Polynesia about 2,000–3,000 years ago (Bellwood, 2011; Skoglund et al., 2016; Gosling and Matisoo-Smith, 2018; Hudjashov et al., 2018; Lipson et al., 2018; Posth et al., 2018). These Austronesians settled in islands like Vanuatu, Tonga, and Samoa for nearly 1,000–2,000 years (Nordyke, 1989; Gosling et al., 2015), where they coinhabited with the Papuan-speaking natives of Northern Melanesia. Today, Polynesian populations [including the Native Hawaiians (Kim et al., 2012)] have varying levels of an ancestry found predominantly in present-day Papuans (Skoglund et al., 2016; Lipson et al., 2018; Posth et al., 2018). The ancient Polynesians began long-range seafaring to the vast stretches of the Pacific around 200 B.C. to 700 A.D., arriving at Hawai‘i between 900 A.D. and 1300 A.D. (Kirch, 1985; Bellwood, 1987; Nordyke, 1989). Inter-island interactions were initially frequent but ceased by the 1400s perhaps due to the development of more complex sociopolitical structures. Native Hawaiians then became relatively isolated until the European settlers arrived (Nordyke, 1989; Gosling et al., 2015). Records of Native Hawaiian population sizes pre-European contact are unreliable, but the effective population sizes (N_*e*_) for Native Hawaiians are likely small throughout history since a genetically estimated N_*e*_ as recent as 1,000 years ago was reported to be ∼1,000 for Melanesians and Samoans (Bergström et al., 2020; Harris et al., 2020). Thus, the demographic history of the Native Hawaiians is likely characterized by multiple founding events and persistent small sizes, which would permit rare alleles to drift to higher frequencies and contribute uniquely to the genetic architecture. Like previous examples from Sardinia, Peru, and Samoa (Sidore et al., 2015; Zoledziewska et al., 2015; Minster et al., 2016; Asgari et al., 2020), a moderate-sized cohort of Native Hawaiians and other Polynesians could provide power to detect these population-specific associations.

**FIGURE 1:**
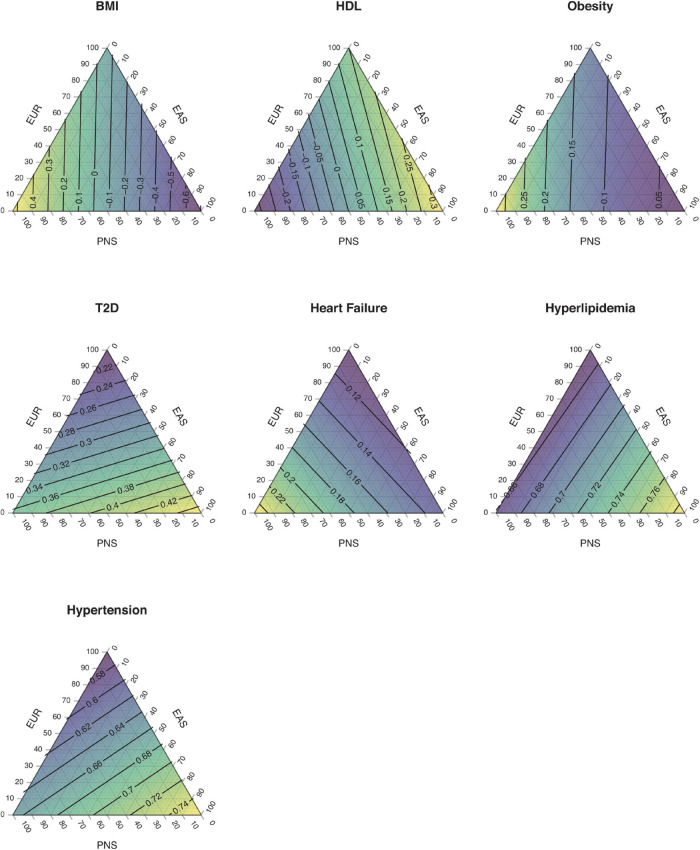
Impact of ancestry components on complex traits and disease risks in Native Hawaiians. The distribution of estimated disease risk are shown as a function of a three-component ancestry model. The linear models used were described in Sun et al. (2021), where for each trait examined as the dependent variable, the effect sizes of the relevant independent variables (e.g., age, BMI, and estimated genetic ancestry as scalar variables, or education level as the categorical variable) were estimated from a Native Hawaiian cohort. Quantitative (BMI and HDL) traits were modeled using linear regression, which predicts the estimated trait value in units of standard deviations given the genetic ancestries. Binary [obesity, type 2 diabetes (T2D), heart failure, hyperlipidemia, and hypertension] traits were modeled using logistic regression, which predicts the probability of disease given genetic ancestries and other covariates. An adult male with age = 50 years, BMI = 30 units (excluded from the obesity model), and education level = college graduate was assumed for calculating probability of disease or estimated trait value. For simplicity, a three-component ancestry model with contributions only from European (EUR), East Asian (EAS), and Polynesian (PNS) ancestors was assumed for Native Hawaiians. The predicted values were interpolated across all possible combinations of ancestries and shown with contour lines. For example, a hypothetical individual with 80% PNS ancestry, 10% EAS, and 10% EUR ancestry aged 50 years, with BMI 30 and college degree, is predicted to have 35–36% chance of being affected with T2D. Similarly, someone with 10% PNS ancestry, 80% EAS, and 10% EUR ancestry of the same age, BMI, and education level is predicted to have ∼42% chance of being affected with T2D. Risk for T2D in Native Hawaiians increases with both PNS and EAS components of ancestry. Note that genetic ancestry captures both genetic and correlated environmental/cultural effects.

Native Hawaiians are also recently admixed. The largest wave of migrants occurred following Captain James Cook’s arrival in Hawai‘i in 1778. Immigrants and missionaries from Europe and Americas as well as laborers from China and East Asia arrived throughout the 19th and 20th centuries. African-ancestry individuals began arriving on the island in the 20th century, mostly as part of the military force (Nordyke, 1989). Today, Native Hawaiians are the group most likely to report having two or more components of ancestry in the United States census (Humes et al., 2011), deriving major continental ancestry from the Polynesians, Europeans, and East Asians (Sun et al., 2021). Variations of these continental ancestries would also partly explain risks of diseases in Native Hawaiians. For example, an individual’s proportion of Polynesian ancestry is associated with the risk of obesity, while both Polynesian and East Asian ancestries contribute to the risk of type 2 diabetes (T2D) (Sun et al., 2021; Figure 1). Note that Polynesian ancestry here is better considered as the component that spread across Polynesia from the initial settlements in remote Oceania. This component itself may be a mixture of the ancient Austronesians that showed close affinity to the East Asian ancestry, as well as the component ancestry native to Melanesia and found predominantly in Papuans today (Gosling et al., 2015; Skoglund et al., 2016). Moreover, while the associations of disease risks with Polynesian ancestry suggest the presence of Polynesian-specific genetic risk factors, the associations are also likely to reflect any cultural or environmental non-genetic factors correlated with Polynesian ancestry (e.g., diet). Nevertheless, past admixture events suggest that approaches such as admixture mapping (Winkler et al., 2010; Shriner, 2017) could identify regions of the genome disproportionately impacting the health of Native Hawaiians.

## Potential Role of Adaptation in Shaping the Genetic Architecture

Adaptive events likely shaped the genetic architecture of complex traits in Native Hawaiians. The successful settlement of previously uninhabited Hawaiian archipelago likely involved adopting new subsistence strategies and overcoming famines, nutritional deficiencies, and higher tropical load of infections (Gosling et al., 2015). The encounter in the 18th century with Europeans and their pathogens deeply impacted the Native Hawaiians: historians have suggested that pathogens such as syphilis, gonorrhea, measles, whooping cough, mumps, cholera, or smallpox, among others, contributed to up to an 80% decrease in census size in Hawai‘i between 1780 and 1850 (Nordyke, 1989). Diets and pathogens are well-known evolutionary forces that shaped the human genome and contributed to phenotypic differences between populations today (Fan et al., 2016; Mathieson, 2020; Rees et al., 2020). As such, adaptation, whether due to forces of nature or actions of the people, could also leave a lasting imprint on the health of Native Hawaiians. However, this hypothesis has not been systematically tested in Native Hawaiians or any Polynesian populations.

Native Hawaiians, and Polynesian populations at large, are more susceptible to metabolic diseases such as obesity and type 2 diabetes (Maskarinec et al., 2009, 2016; Madan et al., 2012; Gosling et al., 2015; Minster et al., 2016; Sun et al., 2021). One contested explanation for this elevated susceptibility is the “Thrifty Gene Hypothesis,” which stipulates that efficient energy storage during times of famine in the past provided an evolutionary advantage that is no longer consistent with the present-day diets. This hypothesis could explain the higher burden of metabolic diseases observed in Polynesian populations today, but there are questions of whether the diversity of environments and genetic ancestries across the Pacific populations would all converge on the same manifestation of risk for metabolic syndromes (Gosling et al., 2015). Genetic support for the Thrifty Gene Hypothesis in other populations has been inconclusive (Ayub et al., 2014; Koh et al., 2014). Results from recent genomic data from Polynesian populations have also been inconsistent, though generally based on single or a few loci (Cadzow et al., 2016; Minster et al., 2016; Lin et al., 2020). Therefore, it is difficult to ascribe the hypothesized selective pressure to the genetic evidence of adaptation. Ultimately, the Thrifty Gene Hypothesis is just one possible reason for adaptation. The focus is not testing the Thrifty Gene Hypothesis, *per se*, but to understand the link between past adaptation and present-day health. Given the advancement in population genetic methods to detect selection across different time scales (Field et al., 2016; Palamara et al., 2018; Edge and Coop, 2019; Speidel et al., 2019), and the emerging genomic data from large epidemiological cohorts from Polynesian populations (Minster et al., 2016; Sun et al., 2021), there is now an opportunity to systematically survey the genome for signature of adaptation and assess their modern-day health consequences.

## Challenges in Genomic Studies With Native Hawaiians

One deterrent to including Native Hawaiians in genomic studies is the underdevelopment of genomic resources. For other continental populations, these resources have been abundant and publicly available, enabling large-scale collaborations and investigations. Development of these resources in Native Hawaiians or other Polynesian populations will similarly accelerate genetic research in these populations.

One sorely needed resource is a catalog of genetic variation, akin to gnomAD, which contains variation discovered from sequencing data of up to ∼141,000 individuals (Karczewski et al., 2020). This catalog has substantially improved clinicians’ ability to interpret clinical sequencing data of severe and rare genetic diseases and to reach a genetic diagnosis. Though still dominated by genomic data from European individuals, gnomAD does include data from ∼20,000 individuals of African ancestry, and similar catalogs are emerging from Asians as well (Chiang et al., 2018; Liu et al., 2018; GenomeAsia100K Consortium, 2019)^1^. However, Native Hawaiians, or Polynesians in general, are not yet represented in these catalogs. The publicly available sequencing data of Native Hawaiians are limited to data from a single individual in the Simons Genome Diversity Project (Mallick et al., 2016). [There are also ∼28 individuals across Oceania in the Human Genome Diversity Panel (Bergström et al., 2020).] Going forward, the sample size need not be large – even a few hundred individuals will allow one to detect nearly all common variations (with frequency >1%) in the population. Since many of these variants will be Polynesian-specific and have not been observed elsewhere in the world, such a catalog will further improve physicians’ ability to interpret variants of unknown significance in the clinical setting to directly benefit the Polynesian community (Easteal et al., 2020).

**FIGURE 2:**
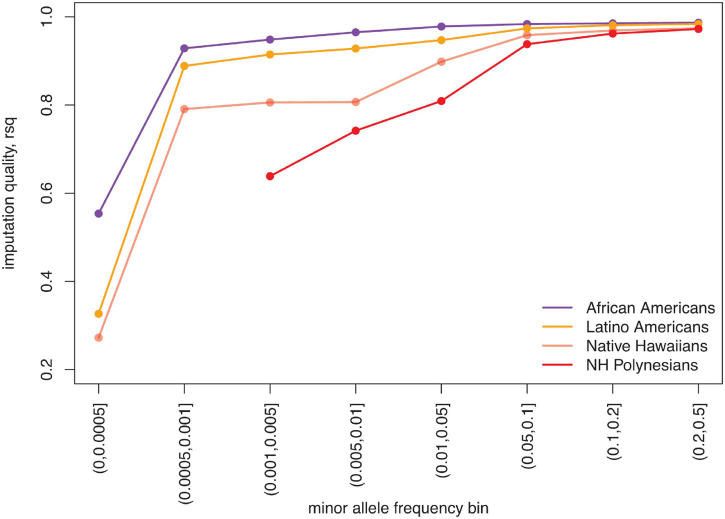
Relatively poor imputation quality for Native Hawaiians due to underrepresentation in imputation reference panels. We imputed 5,325 African Americans, 2,838 Latino Americans, and 3,940 Native Hawaiians from the Multiethnic Cohort (Kolonel et al., 2000) using freeze 8 of the TOPMED imputation server (Taliun et al., 2021) (imputed in July 2020). Each population was genotyped on the MEGA array and subjected to the same QC filters. As measured by the mean imputation quality, R^2^ (rsq), Native Hawaiian individuals are imputed more poorly than other United States ethnic minority populations, particularly for variants with minor allele frequency <5%. The disparity is even stronger when focusing on only the 178 Native Hawaiians with estimated Polynesian ancestry >90% (NH Polynesians) (Lin et al., 2020).

To accelerate the discovery of genetic associations to diseases, we also need to improve Native Hawaiian representation in imputation reference panels. Genome-wide genotyping followed by imputation of the unobserved genetic variation is one of the most efficient approaches to conduct genetic association studies. Publicly available imputation reference panels are constantly growing in size, allowing investigators to query rarer variations that are usually absent on genotyping arrays. Because of the lack of representation in imputation reference panels, the quality of imputation in Native Hawaiians lags significantly behind that of other ethnic minorities (Figure 2). In a proof-of-principle study, it was shown that rs373863828 in *CREBRF* is associated with a large effect on BMI and T2D in Native Hawaiians, but could not be imputed or discovered using publicly available imputation resources at the time, despite the study having sufficient statistical power to do so (Lin et al., 2020). The lack of representation has thus contributed to the disparity in bringing genomic medicine to Native Hawaiians compared to other ethnic minorities in the United States.

Ultimately, larger cohorts will boost statistical power and undoubtedly enhance the genomic insights we can garner, but large recruitments in indigenous communities such as the Native Hawaiians have been challenging. The population sizes of any indigenous population are already small, and past mistakes by researchers, such as the Havasupai diabetes study that misused genetic information from the indigenous community in unconsented studies (Garrison et al., 2019), have also caused community mistrust in scientists. In a recent assessment of Pacific Islanders, over 65% of participants shared some reservation or reluctance about providing biospecimens for research, citing concerns due to spirituality, lack of knowledge of research, or invasion of privacy, among others (Kwan et al., 2015). With increasing awareness of these past mistakes, genome scientists should open dialog with the community early and often, respect both community and individual consent, and *partner with indigenous communities* rather than just enrolling them as participants (Claw et al., 2018; Garrison et al., 2019; Hudson et al., 2020).

## Discussion

Population genetic theories predict the existence of unique genetic variants segregating in the Native Hawaiian population that disproportionately impact their health. Identifying these variants could significantly improve healthcare practices and directly benefit this community. Though several challenges currently exist, the outlook for genetic research in Native Hawaiians and other diverse populations in general can be promising while requiring only a moderate level of funding commitments. Whole genome sequencing of only 150–200 Native Hawaiian individuals would already allow better imputation of Native Hawaiian individuals in a genetic study and accelerate the discovery of population-specific alleles of large effects (Jewett et al., 2012; Lin et al., 2020). The generation and aggregation of WGS data from multiple Polynesian populations will also provide the catalog of genetic variation currently lacking in Polynesian populations, make an immediate impact in the clinical care of Polynesian populations, and accelerate future large-scale genomic research in these populations. Deploying low-coverage sequencing as an alternative first step could also efficiently identify population-specific alleles (Sidore et al., 2015; Chiang et al., 2018; Martin et al., 2021). Importantly, this roadmap is cost-efficient, achievable by pooling resources from a handful of research labs. These are realistic outlooks over the next 5 years.

However, it is important to develop the partnership of the indigenous community in order for the research to proceed. Past exploitation of indigenous populations (Claw et al., 2018; Garrison et al., 2019; Hudson et al., 2020) and the lack of benefits sharing from lucrative pharmaceutical enterprises (Fox, 2020) have brooded mistrust between underprivileged communities and scientists. Research with the indigenous community must also have the community benefits in mind. Note that as health disparity between populations is also driven by non-genetic or social factors, the health benefits derived directly from genomic studies, if any, will likely be slow and not immediately apparent. Nevertheless, it is still important for genomic research to be inclusive if we want to achieve equity and representation; in fact, exclusion of a group of people from research may contribute to inequity in itself. In this context, it is often beneficial for research to be led by scientists of the indigenous community as they are more knowledgeable of the local cultural practices. Alas, there is a dearth of indigenous researchers in the specific research domain described here (see Popejoy and Fullerton, 2016; Merriman and Wilcox, 2018). Whereas pharmaceutical or biotech companies are positioned to directly benefit indigenous communities through proceeds distributions or profit sharing, individual researchers, including non-indigenous ones, are positioned to tailor their engagement to the unique circumstances of each community. By leveraging their long-term individualized interactions, individual researchers will be able to engage in outreach and develop improved and informed consent process, act in stewardship of indigenous data, and help build research capacity through training of the indigenous scientists.

Working within the framework of the Multiethnic Cohort (Kolonel et al., 2000) study, every one of my research projects with – and generally all research proposals utilizing biospecimen data from – the Native Hawaiian population is reviewed by the Native Hawaiian Community Advisory Board (NHCAB) composed of scholars and advocates from the community. A recent study from my group investigating the impact of genetic ancestry on risk of disease in Native Hawaiians (Sun et al., 2021) exemplifies how dialog with community representatives provided the appropriate cultural context. In this study, we observed that the Polynesian component of genetic ancestry (sometimes also with the East Asian component) is associated with risk to certain cardiometabolic diseases (Sun et al., 2021). Through constructive comments from the NHCAB on the early drafts of the manuscript, we came to appreciate that even though the quantification of components of genetic ancestries is a common first step to dissect population-specific genetic risk factors, it should not supplant current approaches (e.g., self-identification or genealogical records) to define community membership. As researchers, we are aware of the deficiency of research methods. We knew that estimated ancestry proportions can be sensitive to the choice of variants analyzed or reference panels used (Uren et al., 2020). We also understood the conceptual difference between genetic ancestry and genealogical ancestry. That is, an individual may not inherit any genetic material from a genealogical ancestor (Donnelly, 1983). But we did not necessarily appreciate how an estimated quantity for research use could detract from an individual’s cultural identity or heritage. It is through communication with the NHCAB that we stressed and repeatedly clarified this concept in our eventual manuscript, and the reviewers noticed.

This is but the first step of active community engagement. A step toward the right direction, but the efforts need to be broadened and made consistent. The Aotearoa New Zealand genomic variome project (Caron et al., 2020) is an example of an inclusive framework in Polynesian populations that others can borrow. The Multiethnic Cohort has been entrusted by >5,000 self-identified Native Hawaiians who donated their biospecimen for research. These individuals have continued to show their support for research by responding to follow-up questionnaires, suggesting that the community is clearly open to partake in research. Now it is up to individual researchers, indigenous or non-indigenous alike, to continue to earn the trust from the indigenous community and be an ally.

## Data Availability Statement

Datasets analyzed in this can be found here in dbGAP with accession number phs000220.v2.p2.

## Ethics Statement

The studies involving human participants were reviewed and approved by the Institutional Review Boards of the University of Hawai‘i and the University of Southern California. The patients/participants provided their written informed consent to participate in this study.

## Author Contributions

CWKC conceived and designed the study, performed the analysis, and wrote the manuscript.

## Conflict of Interest

The author declares that the research was conducted in the absence of any commercial or financial relationships that could be construed as a potential conflict of interest.

## Publisher’s Note

All claims expressed in this article are solely those of the authors and do not necessarily represent those of their affiliated organizations, or those of the publisher, the editors and the reviewers. Any product that may be evaluated in this article, or claim that may be made by its manufacturer, is not guaranteed or endorsed by the publisher.

## Footnotes

1 Genome Medical alliance Japan Project. A Comprehensive Japanese Genetic Variation Database. Available Online at: https://togovar.biosciencedbc.jp/

## References

[B1] Asgari S. Luo Y. Akbari A. Belbin G. M. Li X. Harris D. N. (2020). A positively selected FBN1 missense variant reduces height in Peruvian individuals. *Nature* 582 234–239. 10.1038/s41586-020-2302-0 32499652 PMC7410362

[B2] Ayub Q. Moutsianas L. Chen Y. Panoutsopoulou K. Colonna V. Pagani L. (2014). Revisiting the thrifty gene hypothesis via 65 loci associated with susceptibility to type 2 diabetes. *Am. J. Hum. Genet.* 94 176–185. 10.1016/j.ajhg.2013.12.010 24412096 PMC3928649

[B3] Bellwood P. (2011). Holocene Population History in the Pacific Region as a Model for Worldwide Food Producer Dispersals. *Curr. Anthropol.* 52 S363–S378. 10.1086/658181

[B4] Bellwood P. S. (1987). *The Polynesians: Prehistory of an Island People. Rev. Ed.* London: Thames and Hudson.

[B5] Bergström A. McCarthy S. A. Hui R. Almarri M. A. Ayub Q. Danecek P. (2020). Insights into human genetic variation and population history from 929 diverse genomes. *Science* 367:eaay5012. 10.1126/science.aay5012 32193295 PMC7115999

[B6] Braden K. W. Nigg C. R. (2016). Modifiable Determinants of Obesity in Native Hawaiian and Pacific Islander Youth. *Hawaii J. Med. Public Health* 75 162–171. 27413626 PMC4928515

[B7] Cadzow M. Merriman T. R. Boocock J. Dalbeth N. Stamp L. K. Black M. A. (2016). Lack of direct evidence for natural selection at the candidate thrifty gene locus, PPARGC1A. *BMC Med. Genet.* 17:80. 10.1186/s12881-016-0341-z 27846814 PMC5111290

[B8] Caron N. R. Chongo M. Hudson M. Arbour L. Wasserman W. W. Robertson S. (2020). Indigenous Genomic Databases: pragmatic Considerations and Cultural Contexts. *Front. Public Health* 8:111. 10.3389/fpubh.2020.00111 32391301 PMC7193324

[B9] Chiang C. W. K. Mangul S. Robles C. Sankararaman S. A. (2018). Comprehensive Map of Genetic Variation in the World’s Largest Ethnic Group-Han Chinese. *Mol. Biol. Evol.* 35 2736–2750. 10.1093/molbev/msy170 30169787 PMC6693441

[B10] Claw K. G. Anderson M. Z. Begay R. L. Tsosie K. S. Fox K. Garrison N. A. (2018). A framework for enhancing ethical genomic research with Indigenous communities. *Nat. Commun.* 9:2957. 10.1038/s41467-018-05188-3 30054469 PMC6063854

[B11] Donnelly K. P. (1983). The probability that related individuals share some section of genome identical by descent. *Theor. Popul. Biol.* 23 34–63. 10.1016/0040-5809(83)90004-7 6857549

[B12] Easteal S. Arkell R. M. Balboa R. F. Bellingham S. A. Brown A. D. Calma T. (2020). Equitable Expanded Carrier Screening Needs Indigenous Clinical and Population Genomic Data. *Am. J. Hum. Genet.* 107 175–182. 10.1016/j.ajhg.2020.06.005 32763188 PMC7413856

[B13] Edge M. D. Coop G. (2019). Reconstructing the History of Polygenic Scores Using Coalescent Trees. *Genetics* 211 235–262. 10.1534/genetics.118.301687 30389808 PMC6325695

[B14] Fan S. Hansen M. E. Lo Y. Tishkoff S. A. (2016). Going global by adapting local: a review of recent human adaptation. *Science* 354 54–59. 10.1126/science.aaf5098 27846491 PMC5154245

[B15] Fay J. C. (2013). Disease consequences of human adaptation. *Appl. Transl. Genom.* 2 42–47. 10.1016/j.atg.2013.08.001 27896054 PMC5121272

[B16] Field Y. Boyle E. A. Telis N. Gao Z. Gaulton K. J. Golan D. (2016). Detection of human adaptation during the past 2000 years. *Science* 354 760–764. 10.1126/science.aag0776 27738015 PMC5182071

[B17] Fox K. (2020). The Illusion of Inclusion - The “All of Us” Research Program and Indigenous Peoples’ DNA. *N. Engl. J. Med.* 383 411–413. 10.1056/NEJMp1915987 32726527

[B18] Garrison N. A. Hudson M. Ballantyne L. L. Garba I. Martinez A. Taualii M. (2019). Genomic Research Through an Indigenous Lens: understanding the Expectations. *Annu. Rev. Genomics Hum. Genet.* 20 495–517. 10.1146/annurev-genom-083118-015434 30892943

[B19] GenomeAsia100K Consortium (2019). The GenomeAsia 100K Project enables genetic discoveries across Asia. *Nature* 576 106–111. 10.1038/s41586-019-1793-z 31802016 PMC7054211

[B20] Gosling A. L. Buckley H. R. Matisoo-Smith E. Merriman T. R. (2015). Pacific Populations, Metabolic Disease and “Just-So Stories”: a Critique of the “Thrifty Genotype” Hypothesis in Oceania. *Ann. Hum. Genet.* 79 470–480. 10.1111/ahg.12132 26420513

[B21] Gosling A. L. Matisoo-Smith E. A. (2018). The evolutionary history and human settlement of Australia and the Pacific. *Curr. Opin. Genet. Dev.* 53 53–59. 10.1016/j.gde.2018.06.015 30029008

[B22] Grandinetti A. Chen R. Kaholokula J. K. Yano K. Rodriguez B. L. Chang H. K. (2002). Relationship of blood pressure with degree of Hawaiian ancestry. *Ethn. Dis.* 12 221–228. 12019931

[B23] Grarup N. Moltke I. Andersen M. K. Bjerregaard P. Larsen C. V. L. Dahl-Petersen I. K. (2018a). Identification of novel high-impact recessively inherited type 2 diabetes risk variants in the Greenlandic population. *Diabetologia* 61 2005–2015. 10.1007/s00125-018-4659-2 29926116 PMC6096637

[B24] Grarup N. Moltke I. Andersen M. K. Dalby M. Vitting-Seerup K. Kern T. (2018b). Loss-of-function variants in ADCY3 increase risk of obesity and type 2 diabetes. *Nat. Genet.* 50 172–174. 10.1038/s41588-017-0022-7 29311636 PMC5828106

[B25] Greaves M. (2007). Darwinian medicine: a case for cancer. *Nat. Rev. Cancer* 7 213–221. 10.1038/nrc2071 17301845

[B26] Harris D. N. Kessler M. D. Shetty A. C. Weeks D. E. Minster R. L. Browning S. (2020). Evolutionary history of modern Samoans. *Proc. Natl. Acad. Sci. U. S. A.* 117 9458–9465. 10.1073/pnas.1913157117 32291332 PMC7196816

[B27] Hirschhorn J. N. Daly M. J. (2005). Genome-wide association studies for common diseases and complex traits. *Nat. Rev. Genet.* 6 95–108. 10.1038/nrg1521 15716906

[B28] Hudjashov G. Endicott P. Post H. Nagle N. Ho S. Y. W. Lawson D. J. (2018). Investigating the origins of eastern Polynesians using genome-wide data from the Leeward Society Isles. *Sci. Rep.* 8:1823. 10.1038/s41598-018-20026-8 29379068 PMC5789021

[B29] Hudson M. Garrison N. A. Sterling R. Caron N. R. Fox K. Yracheta J. (2020). Rights, interests and expectations: indigenous perspectives on unrestricted access to genomic data. *Nat. Rev. Genet.* 21 377–384. 10.1038/s41576-020-0228-x 32251390

[B30] Humes K. R. Jones N. A. Ramirez R. R. (2011). *Overview of Race and Hispanic Origin: 2010. 2011 [cited 29 Oct 2020].* Available Online at: https://www.census.gov/library/publications/2011/dec/c2010br-02.html (accessed October 29, 2020).

[B31] Jewett E. M. Zawistowski M. Rosenberg N. A. Zöllner S. (2012). A coalescent model for genotype imputation. *Genetics* 191 1239–1255. 10.1534/genetics.111.137984 22595242 PMC3416004

[B32] Karczewski K. J. Francioli L. C. Tiao G. Cummings B. B. Alföldi J. Wang Q. (2020). The mutational constraint spectrum quantified from variation in 141,456 humans. *Nature* 581 434–443. 10.1038/s41586-020-2308-7 32461654 PMC7334197

[B33] Kim S. K. Gignoux C. R. Wall J. D. Lum-Jones A. Wang H. Haiman C. A. (2012). Population genetic structure and origins of Native Hawaiians in the multiethnic cohort study. *PLoS One* 7:e47881. 10.1371/journal.pone.0047881 23144833 PMC3492381

[B34] Kirch P. V. (1985). *Feathered gods and fishhooks: an introduction to Hawaiian archaeology and prehistory.* Honolulu: University of Hawaii Press.

[B35] Koh X.-H. Liu X. Teo Y.-Y. (2014). Can evidence from genome-wide association studies and positive natural selection surveys be used to evaluate the thrifty gene hypothesis in East Asians? *PLoS One* 9:e110974. 10.1371/journal.pone.0110974 25337808 PMC4206456

[B36] Kolonel L. N. Henderson B. E. Hankin J. H. Nomura A. M. Wilkens L. R. Pike M. C. (2000). A multiethnic cohort in Hawaii and Los Angeles: baseline characteristics. *Am. J. Epidemiol.* 151 346–357. 10.1093/oxfordjournals.aje.a010213 10695593 PMC4482109

[B37] Kwan P. Briand G. Lee C. Lepule J. Llave K. Pang K. (2015). Reservations to Participate in Biospecimen Research among Pacific Islanders. *Calif. J. Health Promot.* 13 27–33. 29805326 PMC5966275

[B38] Lim E. T. Wurtz P. Havulinna A. S. Palta P. Tukiainen T. Rehnstrom K. (2014). Distribution and medical impact of loss-of-function variants in the Finnish founder population. *PLoS Genet.* 10:e1004494. 10.1371/journal.pgen.1004494 25078778 PMC4117444

[B39] Lin M. Caberto C. Wan P. Li Y. Lum-Jones A. Tiirikainen M. (2020). Population-specific reference panels are crucial for genetic analyses: an example of the CREBRF locus in Native Hawaiians. *Hum. Mol. Genet.* 29 2275–2284. 10.1093/hmg/ddaa083 32491157 PMC7399533

[B40] Lipson M. Skoglund P. Spriggs M. Valentin F. Bedford S. Shing R. (2018). Population Turnover in Remote Oceania Shortly after Initial Settlement. *Curr. Biol.* 28 1157–1165.e7. 10.1016/j.cub.2018.02.051 29501328 PMC5882562

[B41] Liu S. Huang S. Chen F. Zhao L. Yuan Y. Francis S. S. (2018). Genomic Analyses from Non-invasive Prenatal Testing Reveal Genetic Associations, Patterns of Viral Infections, and Chinese Population History. *Cell* 175 347–359.e14. 10.1016/j.cell.2018.08.016 30290141

[B42] Locke A. E. Steinberg K. M. Chiang C. W. K. Service S. K. Havulinna A. S. Stell L. (2019). Exome sequencing of Finnish isolates enhances rare-variant association power. *Nature* 572 323–328. 10.1038/s41586-019-1457-z 31367044 PMC6697530

[B43] Lohmueller K. E. (2014). The impact of population demography and selection on the genetic architecture of complex traits. *PLoS Genet.* 10:e1004379. 10.1371/journal.pgen.1004379 24875776 PMC4038606

[B44] Madan A. Archambeau O. G. Milsom V. A. Goldman R. L. Borckardt J. J. Grubaugh A. L. (2012). More than black and white: differences in predictors of obesity among Native Hawaiian/Pacific Islanders and European Americans. *Obesity* 20 1325–1328. 10.1038/oby.2012.15 22286530 PMC3346845

[B45] Mallick S. Li H. Lipson M. Mathieson I. Gymrek M. Racimo F. (2016). The Simons Genome Diversity Project: 300 genomes from 142 diverse populations. *Nature* 538 201–206. 10.1038/nature18964 27654912 PMC5161557

[B46] Martin A. R. Atkinson E. G. Chapman S. B. Stevenson A. Stroud R. E. Abebe T. (2021). Low-coverage sequencing cost-effectively detects known and novel variation in underrepresented populations. *Am. J. Hum. Genet.* 108 656–668. 10.1016/j.ajhg.2021.03.012 33770507 PMC8059370

[B47] Maskarinec G. Erber E. Grandinetti A. Verheus M. Oum R. Hopping B. N. (2009). Diabetes incidence based on linkages with health plans: the multiethnic cohort. *Diabetes* 58 1732–1738. 10.2337/db08-1685 19258435 PMC2712787

[B48] Maskarinec G. Morimoto Y. Jacobs S. Grandinetti A. Mau M. K. Kolonel L. N. (2016). Ethnic admixture affects diabetes risk in native Hawaiians: the Multiethnic Cohort. *Eur. J. Clin. Nutr.* 70 1022–1027. 10.1038/ejcn.2016.32 27026423 PMC5014576

[B49] Mathieson I. (2020). Human adaptation over the past 40,000 years. *Curr. Opin. Genet. Dev.* 62 97–104. 10.1016/j.gde.2020.06.003 32745952 PMC7484260

[B50] Mau M. K. Sinclair K. Saito E. P. Baumhofer K. N. Kaholokula J. K. (2009). Cardiometabolic health disparities in native Hawaiians and other Pacific Islanders. *Epidemiol. Rev.* 31 113–129. 10.1093/ajerev/mxp004 19531765 PMC2893232

[B51] McCarthy M. I. Abecasis G. R. Cardon L. R. Goldstein D. B. Little J. Ioannidis J. P. (2008). Genome-wide association studies for complex traits: consensus, uncertainty and challenges. *Nat. Rev. Genet.* 9 356–369. 10.1038/nrg2344 18398418

[B52] Merriman T. R. Wilcox P. L. (2018). Cardio-metabolic disease genetic risk factors among Mâori and Pacific Island people in Aotearoa New Zealand: current state of knowledge and future directions. *Ann. Hum. Biol.* 45 202–214. 10.1080/03014460.2018.1461929 29877153

[B53] Minster R. L. Hawley N. L. Su C. T. Sun G. Kershaw E. E. Cheng H. (2016). A thrifty variant in CREBRF strongly influences body mass index in Samoans. *Nat. Genet.* 48 1049–1054. 10.1038/ng.3620 27455349 PMC5069069

[B54] Moltke I. Grarup N. Jorgensen M. E. Bjerregaard P. Treebak J. T. Fumagalli M. (2014). A common Greenlandic TBC1D4 variant confers muscle insulin resistance and type 2 diabetes. *Nature* 512 190–193. 10.1038/nature13425 25043022

[B55] Need A. C. Goldstein D. B. (2009). Next generation disparities in human genomics: concerns and remedies. *Trends Genet.* 25 489–494. 10.1016/j.tig.2009.09.012 19836853

[B56] Nordyke E. C. (1989). *The Peopling of Hawaii*, 2nd Edn. Honolulu: University of Hawaii Press.

[B57] Ohta T. (1973). Slightly deleterious mutant substitutions in evolution. *Nature* 246 96–98. 4585855 10.1038/246096a0

[B58] Palamara P. F. Terhorst J. Song Y. S. Price A. L. (2018). High-throughput inference of pairwise coalescence times identifies signals of selection and enriched disease heritability. *Nat. Genet.* 50 1311–1317. 10.1038/s41588-018-0177-x 30104759 PMC6145075

[B59] Pike M. C. Kolonel L. N. Henderson B. E. Wilkens L. R. Hankin J. H. Feigelson H. S. (2002). Breast cancer in a multiethnic cohort in Hawaii and Los Angeles: risk factor-adjusted incidence in Japanese equals and in Hawaiians exceeds that in whites. *Cancer Epidemiol. Biomarkers Prev.* 11 795–800. 12223421

[B60] Popejoy A. B. Fullerton S. M. (2016). Genomics is failing on diversity. *Nature* 538 161–164. 10.1038/538161a 27734877 PMC5089703

[B61] Posth C. Nägele K. Colleran H. Valentin F. Bedford S. Kami K. W. (2018). Language continuity despite population replacement in Remote Oceania. *Nat. Ecol. Evol.* 2 731–740. 10.1038/s41559-018-0498-2 29487365 PMC5868730

[B62] Rees J. S. Castellano S. Andrés A. M. (2020). The Genomics of Human Local Adaptation. *Trends Genet.* 36 415–428. 10.1016/j.tig.2020.03.006 32396835

[B63] Shriner D. (2017). Overview of Admixture Mapping. *Curr. Protoc. Hum. Genet.* 94 1.23.1–1.23.8. 10.1002/cphg.44 28696560

[B64] Sidore C. Busonero F. Maschio A. Porcu E. Naitza S. Zoledziewska M. (2015). Genome sequencing elucidates Sardinian genetic architecture and augments association analyses for lipid and blood inflammatory markers. *Nat. Genet.* 47 1272–1281. 10.1038/ng.3368 26366554 PMC4627508

[B65] Singh G. K. Lin S. C. (2013). Dramatic Increases in Obesity and Overweight Prevalence among Asian Subgroups in the United States, 1992-2011. *ISRN Prev. Med.* 2013:898691. 10.5402/2013/898691 24967142 PMC4045452

[B66] Skoglund P. Posth C. Sirak K. Spriggs M. Valentin F. Bedford S. (2016). Genomic insights into the peopling of the Southwest Pacific. *Nature* 538 510–513. 10.1038/nature19844 27698418 PMC5515717

[B67] Speidel L. Forest M. Shi S. Myers S. R. (2019). A method for genome-wide genealogy estimation for thousands of samples. *Nat. Genet.* 51 1321–1329. 10.1038/s41588-019-0484-x 31477933 PMC7610517

[B68] Spratt D. E. Chan T. Waldron L. Speers C. Feng F. Y. Ogunwobi O. O. (2016). Racial/Ethnic Disparities in Genomic Sequencing. *JAMA Oncol.* 2 1070–1074. 10.1001/jamaoncol.2016.1854 27366979 PMC5123755

[B69] Stearns S. C. Nesse R. M. Govindaraju D. R. Ellison P. T. (2010). Evolution in health and medicine Sackler colloquium: evolutionary perspectives on health and medicine. *Proc. Natl. Acad. Sci. U. S. A.* 107 1691–1695. 10.1073/pnas.0914475107 20133821 PMC2868294

[B70] Steri M. Orru V. Idda M. L. Pitzalis M. Pala M. Zara I. (2017). Overexpression of the Cytokine BAFF and Autoimmunity Risk. *N. Engl. J. Med.* 376 1615–1626. 10.1056/NEJMoa1610528 28445677 PMC5605835

[B71] Sun H. Lin M. Russell E. M. Minster R. L. Chan T. F. Dinh B. L. (2021). The impact of global and local Polynesian genetic ancestry on complex traits in Native Hawaiians. Lachance J, editor. *PLoS Genet.* 17:e1009273. 10.1371/journal.pgen.1009273 33571193 PMC7877570

[B72] Taliun D. Harris D. N. Kessler M. D. Carlson J. Szpiech Z. A. Torres R. (2021). Sequencing of 53,831 diverse genomes from the NHLBI TOPMed Program. *Nature* 590 290–299. 10.1038/s41586-021-03205-y 33568819 PMC7875770

[B73] Tung W. C. Barnes M. (2014). Heart Diseases Among Native Hawaiians and Pacific Islanders. *Home Health Care Manag. Pract.* 26 110–113. 10.1177/1084822313516125

[B74] Uren C. Hoal E. G. Möller M. (2020). Putting RFMix and ADMIXTURE to the test in a complex admixed population. *BMC Genet.* 21:40. 10.1186/s12863-020-00845-3 32264823 PMC7140372

[B75] Visscher P. M. Wray N. R. Zhang Q. Sklar P. McCarthy M. I. Brown M. A. (2017). 10 Years of GWAS Discovery: biology, Function, and Translation. *Am. J. Hum. Genet.* 101 5–22. 10.1016/j.ajhg.2017.06.005 28686856 PMC5501872

[B76] Winkler C. A. Nelson G. W. Smith M. W. (2010). Admixture mapping comes of age. *Annu. Rev. Genomics Hum. Genet.* 11 65–89. 10.1146/annurev-genom-082509-141523 20594047 PMC7454031

[B77] Zhernakova A. Elbers C. C. Ferwerda B. Romanos J. Trynka G. Dubois P. C. (2010). Evolutionary and functional analysis of celiac risk loci reveals SH2B3 as a protective factor against bacterial infection. *Am. J. Hum. Genet.* 86 970–977. 10.1016/j.ajhg.2010.05.004 20560212 PMC3032060

[B78] Zoledziewska M. Sidore C. Chiang C. W. K. Sanna S. Mulas A. Steri M. (2015). Height-reducing variants and selection for short stature in Sardinia. *Nat. Genet.* 47 1352–1356. 10.1038/ng.3403 26366551 PMC4627578

